# Differential Effects of Drugs Targeting Cancer Stem Cell (CSC) and Non-CSC Populations on Lung Primary Tumors and Metastasis

**DOI:** 10.1371/journal.pone.0079798

**Published:** 2013-11-20

**Authors:** Leyre Larzabal, Nefertiti El-Nikhely, Miriam Redrado, Werner Seeger, Rajkumar Savai, Alfonso Calvo

**Affiliations:** 1 Laboratory of Novel Therapeutic Targets, Division of Oncology, CIMA of the University of Navarra, Pamplona, Spain; 2 Department of Molecular Mechanisms in Lung Cancer, Max Planck Institute for Heart and Lung Research, Bad Nauheim, Germany; University of Chicago, United States of America

## Abstract

Cancer stem cells (CSCs) are thought to be responsible for tumor initiation and recurrence after chemotherapy. Targeting CSCs and non-CSCs with specific compounds may be an effective approach to reduce lung cancer growth and metastasis. The aim of this study was to investigate the effect of salinomycin, a selective inhibitor of CSCs, with or without combination with paclitaxel, in a metastatic model. To evaluate the effect of these drugs in metastasis and tumor microenvironment we took advantage of the immunocompetent and highly metastatic LLC mouse model. Aldefluor assays were used to analyze the ALDH+/− populations in murine LLC and human H460 and H1299 lung cancer cells. Salinomycin reduced the proportion of ALDH+ CSCs in LLC cells, whereas paclitaxel increased such population. The same effect was observed for the H460 and H1299 cell lines. Salinomycin reduced the tumorsphere formation capacity of LLC by more than 7-fold, but paclitaxel showed no effect. In *in vivo* experiments, paclitaxel reduced primary tumor volume but increased the number of metastatic nodules (p<0.05), whereas salinomycin had no effect on primary tumors but reduced lung metastasis (p<0.05). Combination of both drugs did not improve the effect of single therapies. ALDH1A1, SOX2, CXCR4 and SDF-1 mRNA levels were higher in metastatic lesions than in primary tumors, and were significantly elevated in both locations by paclitaxel treatment. On the contrary, such levels were reduced (or in some cases did not change) when mice were administered with salinomycin. The number of F4/80+ and CD11b+ cells was also reduced upon administration of both drugs, but particularly in metastasis. These results show that salinomycin targets ALDH+ lung CSCs, which has important therapeutic effects *in vivo* by reducing metastatic lesions. In contrast, paclitaxel (although reducing primary tumor growth) promotes the selection of ALDH+ cells that likely modify the lung microenvironment to foster metastasis.

## Introduction

Lung cancer is one of the leading causes of mortality worldwide and the most common cause of death from cancer in men and women [1]. Most of lung cancer cases belong to the non-small-cell lung cancer (NSCLC) type (85% of them). The prognosis for more than 60% of patients with NSCLC is poor, partly because advanced stage at diagnosis precludes curative surgery, and partly because medical treatments are ineffective. In 2007, the 5-year survival rates for men and women diagnosed with lung cancer were 16%. Unfortunately, these percentages have not changed substantially over several decades despite significant advances in the diagnosis and therapeutic options [2]. Although the use of targeted therapies for lung cancer has been a breakthrough in cancer research, only a small proportion of patients benefit from them. Therefore, there is a clear need for new therapeutic alternatives for a more efficacious treatment of this tumor type. One new therapeutic avenue that is currently being tested in preclinical experiments for a variety of solid tumors is targeting cancer stem cells (CSCs). The CSCs hypothesis states that CSCs are responsible for tumor initiation, cell survival after therapy, metastatic spread and tumor recurrence [3]. Although recent evidence suggests that human lung cancers, like other tumors, may also harbor CSC populations, identification of human lung CSCs has been hampered by the lack of reliable stem cell markers. Expression and activity of aldehyde dehydrogenases (ALDH) serve as CSC markers in breast [4] and lung [5] tumors. Furthermore, increased ALDH activity has been found in stem cell populations in different tumor types including human multiple myeloma, acute myeloid leukemia, brain, breast, liver, colon, pancreas [6], [7] and, more recently, in lung, where ALDH1A1 expression is associated with poor survival in a cohort of stage I NSCLC patients [8]. The ALDH+ fraction is enriched in tumor initiating cells with increased migration, adhesion ability and metastatic potential [9]. Together, these findings suggest that measurement of ALDH levels or enzymatic activity may serve as a lung CSC marker.

CSCs are resistant to many current cancer treatments, including chemotherapy and radiotherapy [10], [11]. This suggests that many cancer therapies, while killing the bulk of tumor, may ultimately fail because they do not eliminate CSCs, which manage to survive and to regenerate new tumors. Recent experimental evidence also suggests a great plasticity of both CSC and non-CSC populations [12]. This has led some authors to hypothesize that targeting both CSC and non-CSC populations are likely to be necessary for a more effective treatment, although this issue has not been experimentally tested yet.

Salinomycin, a potassium ionophore, was recently identified as a selective inhibitor of human breast cancer stem cells *in vitro* [13]. Although the mechanism of action of salinomycin is not yet clear, it appears to act as a potent inhibitor of the multidrug resistance P-glycoprotein (P-gp/MDR1/ABCB1) [14].

Tumor cell migration and metastasis, as important features of tumor biology share many similarities with leukocyte trafficking, which is critically regulated by chemokines and their receptors. Accumulating data suggest that CXCR4 (CXC chemokine receptor-4) and SDF1 (stroma cell-derived factor-1, or CXCL12) could regulate migration and metastasis in a variety of lung, breast and prostate cancer cells [15]. Moreover, CXCR4 overexpression correlated with poor prognosis in many tumor types [16]. CSCs express CXCR4 receptor and respond to a chemotactic gradient of its specific ligand SDF-1 [17], suggesting that CSCs probably represent a subpopulation capable of initiating metastasis [18]. A better understanding of the migratory mechanism involving cancer cells and CSCs would provide more appropriate tools for developing novel therapies to reduce both local and distant recurrences.

CSCs interact with surrounding non-malignant cells and both cell types are co-regulated within the tumor microenvironment [19]. Cells from the tumor microenvironment not only could enhance growth of the primary tumor but also facilitate its metastatic dissemination to distant organs. Successful metastatic outgrowth thus depends on the ability of cancer cells to take advantage of the surrounding microenvironment at each step of the metastatic process. Tumor microenvironment comprises numerous cells including endothelial cells, stromal fibroblasts and a variety of bone marrow derived cells (BMDCs) such us macrophages, myeloid-derived suppressors cells, Tie2-expressing monocytes and mesenchymal stem cells [20]. When tumor cells arrive to the site of metastasis they have to get adapted to a new microenvironment that is different from that of the primary tumor [21]. The precise tumor-stroma interactions in primary tumor and metastasis site are largely unknown.

Previous seminal work has shown that salinomycin and paclitaxel exhibited distinct modes of action in a mouse model of breast cancer, with salinomycin possessing CSC-specific inhibitory activities [13]. Considering that CSCs may be key mediators of metastasis, we hypothesized that specifically targeting CSCs with salinomycin would reduce the metastatic potential of residual tumor cells. To address this hypothesis, we investigated the efficacy of the presumed CSC-specific drug salinomycin on the growth and metastatic spread of NSCLC. We report here that salinomycin differentially affects the NSCLC primary tumor growth rate and metastatic potential relative to those of the conventional chemotherapeutic agent paclitaxel, biological activities correlating with measures of CSC traits. Also using the Lewis lung carcinoma (LLC) mouse model, we report how these compounds affect the tumor microenvironment in both primary and metastatic tumor sites.

## Materials and Methods

### Cell Culture and Compounds

Cell lines used in this study included mouse Lewis lung carcinoma (LLC) and human H460 and H1299. All cell lines were obtained from the American Type Culture Collection (Manassas, VA, USA) and were maintained in RPMI (Sigma, St Louis, MO, USA) with 10% fetal bovine serum (Thermo Scientific, Waltham, MA, USA) and 1% penicillin-streptomycin (Lonza, Basel, Switzerland), at 37°C and 5% CO_2_ atmosphere. Salinomycin and paclitaxel were obtained from Sigma (St Louis, MO, USA) and were dissolved in 20% DMSO in PBS (vehicle). The final concentrations of salinomycin and paclitaxel used in all *in vitro* assays were 1 µg/mL for salinomycin and 40 ng/mL for paclitaxel. Cells treated with vehicle were used as controls. These doses were used based on previous publications [13].

### Sphere Formation Assay

For sphere formation, cells were grown in serum free culture medium DMEM/F12+ GlutMAX™ (Gibco, Paisley, UK) supplemented with growth factors (MEGM SingleQuots, Lonza, Basel, Switzerland) and B27 (Gibco, Paisley, UK). Cells were plated in 6-well ultra low attachment plates (Corning, Lowell, MA, USA) at a density of 1000–5000 cells per well depending of the cell line and cultured for 7–10 days. To assess the self-renewal potential of these cells, the first generation of spheres was collected by gentle centrifugation, dissociated into single-cell suspensions, and cultured under the conditions described above for another 7 to 10 days. To test the effect of drugs in the sphere formation ability, cells were plated in the presence of 20% DMSO (vehicle), salinomycin (1 µg/mL) or paclitaxel (40 ng/mL) at the beginning of the experiment, without further addition of the drug. After 7 days, plates were analyzed for lung tumorspheres formation and the number of spheres per well was quantified using an inverted microscope (Olympus, Hamburg, Germany). To evaluate the effect of drug treatment in the ALDH+ population in LLC-derived spheres, the formed spheres were treated with paclitaxel (40 ng/mL) or vehicle for 72 hours. After incubation, Aldefluor assays were performed by flow cytometry.

### ALDH Staining and Cell Sorting

The Aldefluor kit (StemCell Technologies, Durham, NC, USA) was used to profile and separate cells with high and low ALDH enzymatic activity. The experiments were undertaken according to the manufacturer’s instruction. Briefly, 1×10^6^ cells were incubated in Aldefluor buffer containing the ALDH protein substrate (BAAA, BODIPY-aminoacetaldehyde, 1 mmol/L) for 30 minutes at 37°C. Cells that could catalyze BAAA to its fluorescent product (BAA) were considered ALDH+. Sorting gates for FACS were drawn relative to cell baseline fluorescence, which was determined by the addition of the ALDH-specific inhibitor diethylaminobenzaldehyde (DEAB) during the incubation. 7-aminoactinomycin D (7-AAD; Sigma-Aldrich) was used to enumerate viable, apoptotic and dead cells (Figure S1A). Cells were sorted in a FACSAria Ilu (BD Biosciences, Franklin Lakes, NJ, USA) and the purity of sorted cells was assayed after the process was completed (Figure S1B). The data were analyzed by Cell Quest Pro (BD Biosciences, Franklin Lakes, NJ, USA) and FlowJo softwares (Ashland, USA).

### CXCR4 Flow Cytometry

After the incubation of LLC cells with vehicle, salinomycin (1 µg/mL) or paclitaxel (40 ng/mL) for 72 hours cells were washed once with PBS and then harvested with 0.05% trypsin/0.025% EDTA. Detached cells were washed with PBS/EDTA/BSA (washing buffer) and resuspended in the washing buffer (10^6^ cells/100 µl). CXCR4 antibody (Sigma) or its respective isotype control was added to the cell suspension at 1∶50 concentration and incubated at 4°C for 30 min. The labelled cells were washed again and the secondary antibody conjugated to FITC (BD Bioscience) was added and incubated at 4°C in darkness for 30 min. Cells were washed and analyzed on a FACSCalibur (BD Biosciences).

### Clonogenic Assay

To evaluate the clonogenic potential of ALDH positive and negative populations of LLC cells after sorting, 500 cells per well were plated into 6-well plates in adherent conditions. After 10 days in culture, colonies were fixed with 4% buffered formalin (Panreac, Barcelona, Spain) and stained with 2% crystal violet. The number of colonies per well was determined.

### Cell Proliferation and Cytotoxicity Assay

Cell proliferation was determined by MTT assay (Roche, Palo Alto, USA). Sorted ALDH positive and negative populations of LLC cells or unsorted LLC cells were seeded in 96-well culture plates (1000 cells per well) in 100 µl of medium. 24, 48, 72 and 96 hours later 10 µl of MTT was added. Spectrophotometric absorbance was measured at 570 nm.

For cytotoxicity assay, LLC cells grown in adherent or in anoikis-resistant (sphere-forming) conditions were plated in 96-well plates (1000 cells per well) and treated with serially diluted paclitaxel (0–100 nM). Seventy-two hours after incubation, MTT assays were performed following the manufacturer’s protocol. The percentage of cell survival was normalized by dividing the final absorbance of treated samples by that of the untreated control, and the IC_50_ was calculated.

### RNA Extraction and Quantitative Real Time PCR (qRT-PCR)

Total RNA was isolated from cells, sphere-containing pellets, or frozen tissue samples using QIAamp RNeasy Minikit (Qiagen, Chatsworth, CA). After DNase I treatment, reverse transcription was performed with Superscript II reverse transcriptase (Invitrogen, Carlsbad, CA) to generate complementary DNA (cDNA). qRT-PCR was run in an Applied Biosystems 7900 Real-time PCR machine. qRT-PCR reactions were carried out with SYBR Green PCR Master Mix (Applied Biosystems, Forster City, CA, USA) and GAPDH levels were used as controls. The mean cycle threshold value (Ct) for the gene of interest, normalized to the Ct value of the housekeeping gene (GAPDH) was used to calculate gene expression values. Assays were performed to quantify mRNA levels of mouse and/or human ALDH1A1, SOX2, CXCR4, SDF-1, CD11b, VEGF and VEGFR1 genes. The primer sequences are shown in Table S1. Data are given as 2^−ΔΔCt^ or 2^−ΔCt^.

### Migration Assay (Boyden Chamber)

Migration assays were conducted in a Boyden chamber. 20000 cells per well in serum-free medium were seeded in the upper transwell of 24-well plates (Costar) in the presence of vehicle, salinomycin (1 µg/mL) or paclitaxel (40 ng/mL). Medium with 10% of serum, used as a chemoattractant, was placed in the lower compartment. After 48 hours, cells in the top chamber were wiped with a cotton swab, and cells in the lower compartment were fixed with 4% formalin and stained with crystal violet. The number of migratory cells was evaluated with a Leica DMIL LED microscope using the LAS EZ software (Leica Microsystems).

### Animal Studies

Animal studies were carried out according to the ethical guidelines established by our Institutions (University of Navarra), under an approved animal protocol by the Committee on the Ethics of Animal Experiments of the University of Navarra (069/11). All animals were housed in microisolator cages and in SPF conditions. All surgery procedures was performed under anesthesia and all efforts were made to minimize suffering of the animals.

For the evaluation of the tumor initiating capability of CSC, six-week-old NSG (NOD SCID IL2Rg mice from The Jackson Laboratory, USA) were used. One thousand or five thousand ALDH positive or negative sorted LLC cells were injected subcutaneously into the flanks of the mice in PBS. Tumor volume was measured after 3 weeks with an electronic caliper. At the end of the experiment animals were sacrificed by CO_2_ inhalation. Four mice per group were used.

To test the effect of salinomycin and paclitaxel *in vivo,* 5×10^5^ LLC cells were injected subcutaneously into the flank of 8-week old C57BL/6 mice. Drug treatment was initiated 6 days after tumor cell injection, time at which the primary tumor reached approximately 50 mm^3^. Animals were administered with either 20% DMSO in PBS (vehicle), salinomycin (5 mg/kg), paclitaxel (5 mg/kg), or a combination of both drugs every 2 days, by intraperitoneal injection, for 5 weeks. These doses and treatment schedules were selected based on previous publications [13]. Tumors were measured every 2 days with an electronic caliper. When primary tumors reached approximately 300 mm^3^, tumors were surgically removed and the incisions were sewn. The operation was performed with anesthetized mice using isoflurane (Esteve, Barcelona, Spain) and in aseptic conditions. In order to reduce the suffering of animals they were administered with ketoprofen (5 mg/kg) every 24 hours for 3 days after surgery. Three weeks later, animals were sacrificed by CO_2_ inhalation and lungs were removed for further analysis of metastatic nodules. Each group comprised 5 mice and the experiment was repeated twice, in order to confirm the results.

### Staining and Image Analysis

Tissue sections were fixed in 4% buffered formalin, embedded in paraffin, and sectioned (5 µm in thickness). Slides were stained with H&E. For lung metastasis quantification, digital images from lung sections from each of the animals (n = 5 per group) were acquired with an AxioPlan 2 microscope (Zeiss, Germany) using an in-house Metamorph macro (Molecular Devices, USA), which allows to compose mosaic images captured at 2.5x, with automatic focus and shadow correction. Images were automatically analyzed with ImageJ and the area occupied by tumor nodules with respect to non-malignant lung area was quantified.

### Immunohistochemistry and Immunofluorescence

To stain mast cells, a solution of toluidine blue (0.1%) was applied to the tissue sections after deparaffinization and rehydration.

For CD31 immunohistochemistry, antigen retrieval was carried out by heating slides in a microwave for 20 min in 10 mM citrate buffer at pH 6. Tissues were then incubated for 1 h at RT with primary anti-CD31 antibody (Dianova, Hamburg, Germany) at 1∶20 dilution. Then, slides were incubated for 30 min at RT with a secondary rabbit anti-rat antibody (Dako) at 1∶50 dilution in Dako Real antibody diluent (Dako). Slides were then incubated for 30 min at RT with the EnVision™ anti-rabbit detection system (Dako). Peroxidase activity was developed with DAB as previously described. Samples were counterstained with haematoxylin, dehydrated and mounted with DPX (VWR, Leicestershire, UK). For quantifications, 150 random images (200x) per experimental group (30 per animal) were captured with a Leica microscope (Wetzlar, Germany) equipped with the Analysis™ software. Positive immunostaining was filtered from the non-stained tissue and quantified with Image J (NIH Image, Bethesda, USA).

For immunofluorescence, tissue sections were hydrated and incubated with trypsin for 10 min for antigen retrieval. Then, tissues were immersed in 5% BSA with 0.1% Triton-X in PBS for 1 h at RT, to avoid unspecific binding of the antibodies. The following primary antibodies were used for immunofluorescent labeling: anti-F4/80 (1∶200; AbD Serotec, Raleigh, North Carolina, USA) and anti-CD11b (1∶100; Abcam, Cambridge, UK). After incubation with the primary antibodies at 4°C overnight, slides were incubated with AlexaFluor 488–labeled secondary antibody (Molecular Probes, Invitrogen, Paisley, UK), counterstained with nuclear 4,6-diamidino-2-phenylindole (DAPI) staining and mounted with Dako fluorescent mounting media (Dako). Quantification of CD11b, F4/80 and toluidine blue positive cells was carried out by computer-aided image analysis, using Leica DMLA and QWin 500IW systems (Leica Instruments). In primary tumors, each specimen was analyzed in 5–10 randomly selected optical fields and the percentage of positive cells with respect to the total number of cells stained with DAPI was calculated. In lung nodules, positive cells were counted manually because we found it more reliable in these types of small lesions, and data were expressed as a percentage of tumor area (mm^2^).

For ALDH1A1 immunostaining, H460 cells were grown in chamber slides (BD Biosciences) and, when confluent, slides were fixed/permeabilized in acetone/methanol 1∶1 for 5 min. Endogenous peroxidase was quenched with a 3% hydrogen peroxide solution and nonspecific binding sites were blocked for 30 min with 5% goat normal serum. Cells were then incubated with anti ALDH1A1 antibody (BD), at 1∶100 dilution, for 2 h at RT and rinsed in TBS. Detection of primary antibody was carried out with the Envision anti-mouse system (Dako, Glostrup, Denmark). Peroxidase activity was developed with DAB (3,3′-diaminobenzidine; Dako) and cells were counterstained with haematoxylin. Finally, slides were cover-slipped with glycerol mounting-medium.

### Statistical Methods

Statistical differences between groups were examined with the Student’s *t* test or ANOVA for unpaired parametric variables, and the Mann-Whitney *U* test or Kruskal-Wallis for unpaired non-parametric variables. Normality was analyzed with the Shapiro-Wilk test. Data were analyzed with the SPSS statistical software (version 17.0 for Windows SPSS) and GraphPad Prism 5 software (GraphPad). Values are expressed as means ± SEM or SD, and statistical significance was defined as P<0.05 (*), P<0.01 (**), and P<0.001 (***).

## Results

### Identification of the ALDH+ CSC-like Population in LLC Cells

**Figure 1:**
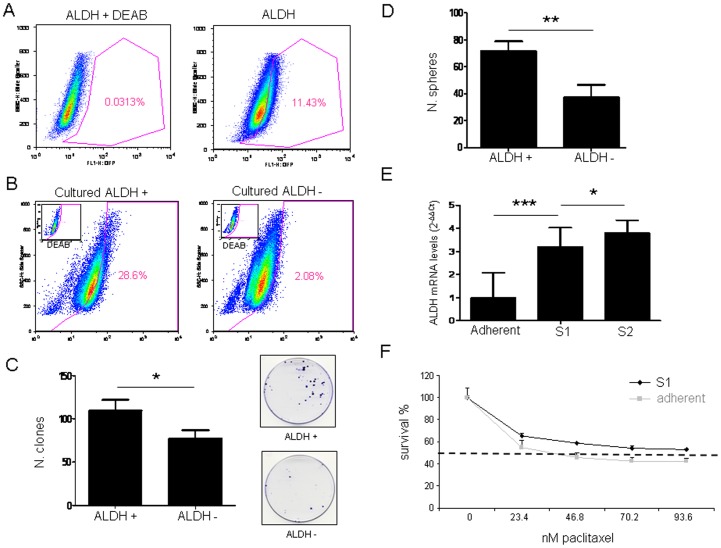
The ALDH+ LLC cell population displays CSC-like characteristics. **A.** The ALDH+ cell fraction (measured by Aldefluor assays) represents ∼11% of total cells in the LLC cell line. **B.** Isolated LLC ALDH+ cells possess self-renewal ability as shown by their capacity to generate both ALDH+/− populations after 8 days in culture, while ALDH− cells only give rise to negative cells. **C.** Number of clones generated by LLC ALDH+ and ALDH− cells after sorting. The ALDH+ population shows higher clonogenic capacity than their negative counterpart. **D.** Number of spheres generated by LLC ALDH+/− cells. **E.** qRT-PCR analysis of ALDH1A1 mRNA levels in LLC cells cultured in adherent conditions, primary spheres (S1) and spheres of second generation (S2). **F.** Chemoresistance to paclitaxel of LLC grown in either adherent conditions or in spheres (S1) was measured with an MTT assay. IC_50_ for adherent LLC cells: 35.8 nM; IC_50_ for LLC S1 spheres: >93.6 nM. Data and error bars: mean ± SD. *p<0.05. **p<0.01. ***p<0.001. All the experiments were repeated at least 3 independent times (each of them in triplicates).

We and others have previously shown that measurement of ALDH activity is an appropriate method for the identification of lung cancer stem cells (CSCs) [22], [23], but whether this marker could be used to isolate and characterize the LLC CSC population was unknown. To assess the presence of this population in the LLC cell line based on their ALDH enzymatic activity, Aldefluor assay followed by FACS analysis were carried out. As shown in Figure 1A, the LLC cell line had an average of 11.43±0.38% ALDH positive cells, which is in keeping with previously published results for other human cell lines and primary cells from patients [5]. The use of DEAB (a specific ALDH inhibitor) served as negative control.

Typical properties of CSC include their capacities for self-renewal and differentiation, *in vivo* tumorigenic potencial and resistance to chemotherapy [24]. To verify these features in LLC cells, we first examined the self-renewal ability of the ALDH+ population. After ALDH cell sorting, both positive and negative cell fractions were plated separately. After 8 days in culture, Aldefluor assay was carried out to analyze the phenotype of the resulting cell population. Cultured ALDH+ cells were able to regenerate both ALDH positive and negative cell populations. On the contrary, cultured ALDH− cells were unable to generate the ALDH+ population (Figure 1B). This result demonstrates that only ALDH+ cells are able to reconstitute the whole cell population, a typical property of CSCs. MTT assays revealed that there were no differences in growth rates between ALDH+ and ALDH− cell populations (Figure S1C). No differences were found between the unsorted parental population and ALDH+ or ALDH− populations (data not show).

To compare the tumorigenic potential of ALDH+ and ALDH− LLC cells, NSG mice were injected subcutaneously with 1×10^3^ or 5×10^3^ cells. As shown in Table 1, tumor growth was observed in 3 of 4 mice after inoculation with 1×10^3^ ALDH+ cells, whereas no tumor growth was observed in the ALDH− cells. After injection of 5×10^3^ ALDH+ cells, 3/4 mice presented a tumor mass with a mean tumor volume of 235.3±64.6 mm^3^, while only 2/4 mice developed a small tumor after injection of the ALDH− population (with a tumor volume of 27.45±5.47 mm^3^).

**Table 1:** Tumour incidence of LLC ALDH+ and ALDH− cells three weeks after cell injection. Four animals per group were used.

No of tumor cells inoculated	ALDH+	ALDH−
1×10^3^	3/4	0/4
5×10^3^	3/4	2/4

We then analyzed the clonogenic potential of the LLC ALDH+ and ALDH− cell fractions by plating 500 cells per well in a 6-well plate and culturing them at clonal density for 10 days. ALDH+ cells gave rise to a significantly higher number of colonies than ALDH− cells (p<0.05; Figure 1C), thus showing that ALDH+ cells display increased clonogenicity. The clonogenic ability of the parental cell line was intermediate between the ALDH+ and ALDH− fractions (data not show).

ALDH+ cells produced a higher number of spheres than ALDH− cells (p<0.01; Figure 1D). We also analyzed the ability of the whole LLC population to form spheres and to self-renew, giving rise to a secondary sphere population (S2). After 7 days in serum and adherent free condition, LLC cells formed primary spheres (S1). Disaggregation of S1 spheres and culture in the same conditions demonstrated the formation of a secondary (S2) sphere population. In order to confirm that spheres were enriched in CSCs, we quantified ALDH mRNA levels in cells grown in adherent and non-adherent (S1 and S2 spheres) conditions. By qRT-PCR analysis we observed that S1 spheres had significantly higher (p<0.001) ALDH mRNA levels than cells cultured in adherent conditions, and that S2 spheres were also enriched in ALDH expression (p<0.05) as compared to S1 spheres (Figure 1E). Furthermore, Aldeflour analysis demonstrated a higher number of ALDH+ cells in spheres (34.2%±6.56%) than in cells grown in adherent conditions (11.43±0.38%) (Suplemmentary Figure 1D and Figure 1A). Therefore, these experiments confirmed that the anoikis-resistant spheres contained an enriched ALDH+ CSC-like population that was able to undergo self-renewal.

Another characteristic of CSCs is their resistance to conventional chemotherapeutic agents. Thus, we used paclitaxel, a commonly used drug against NSCLC, to evaluate chemoresistance of LLC cells grown in spheres or in adherent conditions. As shown in Figure 1F, LLC cells cultured as spheres exhibited higher survival rates when treated with different doses of paclitaxel than cells cultured in adherent conditions. The IC_50_ values for both cell populations were significantly (p<0.01) different: IC_50_ for adherent LLC cells was 38.5 nM and >93.6 nM for LLC-derived spheres (Figure 1F). To address whether S1-derived cells corresponded to the ALDH+ population with increased resistance to paclitaxel, we analyzed by Aldefluor the percentage of ALDH+ cells in spheres following drug treatment. Unexpectedly, we did not observe a significant increase in the percentage of ALDH+ cells in response to paclitaxel in the LLC model in sphere culture conditions. However, as we shall describe below, this phenomena was apparent in human NSCLC models, supporting the premise that S1-derived cells include paclitaxel-resistant ALDH+ cells. Taken together, all these results strongly suggest that the ALDH+ fraction of LLC cells corresponds to a drug resistant CSC population.

### Differential Effect of Salinomycin and Paclitaxel on Targeting CSC vs. Non-CSC Populations

After having demonstrated that the LLC cell line has an ALDH+ sub-population with CSCs features, we wanted to study the effect of salinomycin, a recently identified compound that selectively inhibits human breast cancer stem cells *in vitro* [13]. We compared the effect of salinomycin with that of paclitaxel in CSC vs. non-CSC populations.

**Figure 2:**
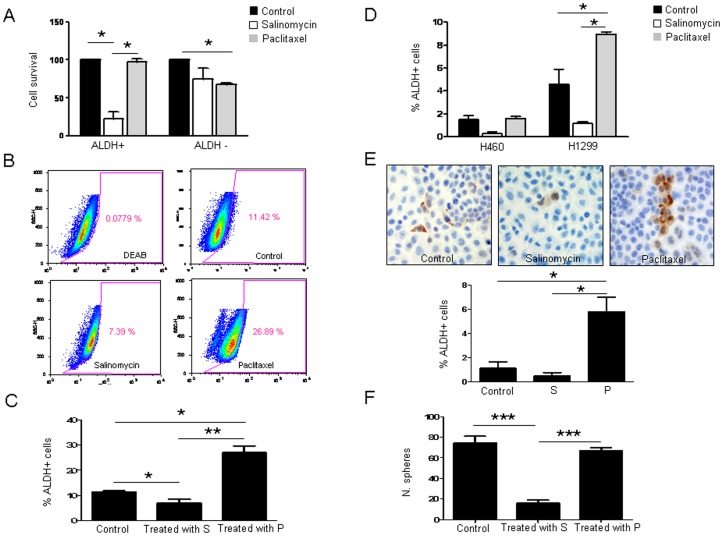
Salinomycin decreases the ALDH positive cell population in LLC cells, whereas paclitaxel increases such population. **A.** Percentage of viable cells after exposure of LLC ALDH+/− cells to vehicle (20% DMSO in PBS) salinomycin (1 µg/mL) or paclitaxel (40 ng/mL) for 72 h after sorting. A strong cytotoxicity is observed for the ALDH+ fraction after salinomycin treatment. In the case of paclitaxel, ALDH+ cells were unaffected, whereas ALDH− cells are growth inhibited. **B–C.** Salinomycin (1 µg/mL) or paclitaxel (40 ng/mL) was administered to the whole LLC unsorted cell population for 72 hours and ALDH+ cells were analyzed by Aldefluor assay. FACS quantifications reveal a reduction in the percentage of ALDH+ cells upon treatment with salinomycin. Treatment with paclitaxel increases the ALDH+ population. **D.** FACS analysis shows similar effects in H460 and H1299 human lung cancer cell lines: salinomycin (1 µg/mL) decreases the ALDH+ population whereas paclitaxel (40 ng/mL) increases such population after 72 hours of treatment. **E.** ALDH1A1 immunostaining (200x) and quantification of ALDH+ cells in the H460 cell line after salinomycin (1 µg/mL) or paclitaxel (40 ng/mL) treatment for 72 hours. **F.** Sphere formation ability upon drug administration. Data and error bars are presented as mean ± SD. S: Salinomycin. P: Paclitaxel. *p<0.05. ***p<0.001. All the experiments were repeated at least 3 independent times (each of them in triplicates).

For subsequent *in vitro* experiments, we treated LLC cells with 1 µg/mL salinomycin or 40 ng/mL paclitaxel, allowing them to recover for 24 hours in absence of the drug. To determine the specific effects of salinomycin and paclitaxel on ALDH+/− cell populations, Aldefluor assays followed by FACS analysis was carried out. After cell sorting, ALDH positive and negative cells were plated separately and administered with salinomycin (1 µg/mL) or paclitaxel (40 ng/mL) for 72 hours and cell viability was assessed by MMT assays. As shown in Figure 2A, survival of ALDH+ cells after salinomycin treatment was significantly lower than that of ALDH− cells (p<0.05). In contrast, after paclitaxel treatment survival of ALDH+ cells was not affected, whereas only 67.7% ALDH− cells were alive (p<0.05). These results show that salinomycin is highly cytotoxic for ALDH+ cells but paclitaxel affects the ALDH negative population.

We also studied the effect of both compounds on the percentage of ALDH+ cells grown in adherent conditions (Figure 2B and C). Salinomycin decreased the percentage of ALDH+ LLC cells in the adherent cell population from 11.42% to 7.39±1.09% (p<0.05) whereas paclitaxel increased this population to 26.89±2.3% (p<0.05). To verify these findings, ALDH mRNA levels were quantified by qRT-PCR. Similarly to results found by flow cytometry, salinomycin reduced ALDH expression, whereas an average of 2.5-fold increase was detected in paclitaxel-treated cells (Figure S2A).

We wanted to corroborate these results in human lung cancer cell lines and Aldefluor assays were performed to study the effect of both drugs in the ALDH+ population in H460 and H1299 cell lines. As shown in Figure 2D and in Figure S2B, paclitaxel increased significantly (p<0.05) the percentage of ALDH+ cells in H1299 whereas salinomycin had an opposite effect, although just beyond the cutoff value for statistical significance (p = 0.06). Similar to our observations in LLC cells, paclitaxel significantly raised ALDH mRNA levels in both H460 and H1299 cells (Figure S2C; p<0.01 and p<0.05, respectively). Although salinomycin treated H460 and H1299 cells exhibited decreased ALDH mRNA levels (0.5-fold and 0.3-fold, respectively), this effect was not statistically significant. Immunocytochemical ALDH analysis of H460 cells further confirmed the presence of a higher number of ALDH+ cells upon paclitaxel treatment (p<0.05) (Figure 2 E).

Levels of SOX2 were measured in drug-treated and control cells as well. SOX2 is a transcription factor essential for the maintenance of self-renewal capacity of undifferentiated embryonic stem cells, which promotes pluripotency [25]. We found that paclitaxel increased (p<0.01) SOX2 levels in the LLC cell line (Figure S2D) whereas salinomycin had no effect. On the contrary, in the human lung cancer cell lines (H460 and H1299), a drop in SOX2 expression was observed after administration of salinomycin (p<0.05). Exposure to paclitaxel had barely any effect on SOX2 expression (Figure S2E).

We next analyzed the effect of both drugs to hinder sphere formation. As shown in Figure 2F, salinomycin diminished dramatically (p<0.001) the sphere formation ability of LLC cells, whereas the number of spheres was similar to controls when cells were exposed to paclitaxel. A similar effect was found in the human lung cancer cell lines. Salinomycin inhibited sphere formation in H460 (p<0.001) and H1299 (p<0.01) cell lines but paclitaxel had no effect (Figure S2F).

Taken together, all these *in vitro* results suggested that paclitaxel affected the non-CSC fraction but selected CSCs. On the contrary, salinomycin decreased the CSC population and inhibited CSCs features.

### Differential Effects of Salinomycin and Paclitaxel on Tumour Growth and Metastasis

Because CSCs have been suggested as metastasis-initiating cells [4], [9] and, as shown by our results, salinomycin targets ALDH+ CSCs, we hypothesized that salinomycin could prevent primary metastasis *in vivo*. To test this hypothesis, we took advantage of the immunocompetent and highly metastatic LLC mouse model. To this end, LLC cells were first injected subcutaneously in C57/Bl6 mice and 6 days after cell injection (when tumor volume reached an approximate size of 50 mm^3^), animals were administered with either vehicle, paclitaxel (5 mg/kg), salinomycin (5 mg/kg), or a combination of both drugs. When primary tumors reached an approximate volume of 300 mm^3^, tumors were surgically removed and mice were allowed to develop lung metastasis for the following 3 weeks.

**Figure 3:**
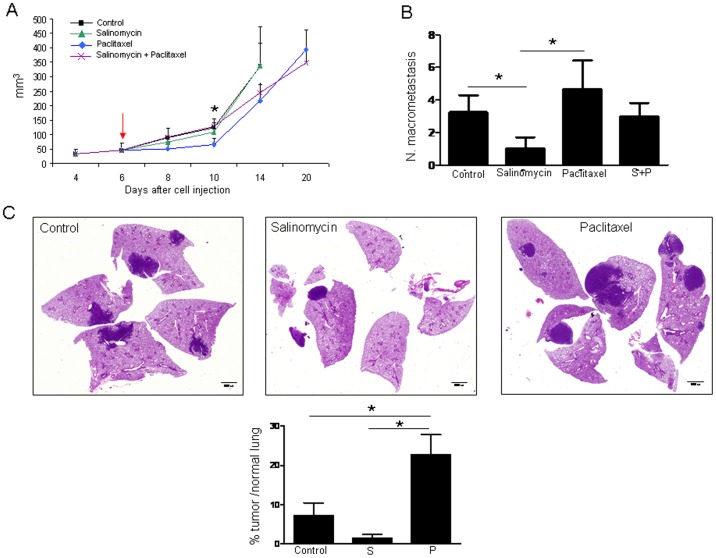
Effect of salinomycin and paclitaxel on primary tumor growth and lung metastasis development. **A.** Primary tumor volumes. Paclitaxel delays tumor growth compared with control mice. The arrow indicates the day of treatment initiation. **B.** Macroscopic evaluation of the number of lung macrometastasis. Mice administered with salinomycin display a reduction in the number of lung macrometastatic foci compared with control mice. Animals that received paclitaxel show a higher number of macrometastasis compared with salinomycin treated mice. **C.** Representative histological images of lungs from control mice and salinomycin or paclitaxel injected mice. Quantification of metastasis by image analysis in histological sections. Data are expressed as percentage of area occupied by metastatic nodules with respect to area of the normal lung. Data and error bars show mean ± SEM. 5 animals per group were used. S: Salinomycin. P: Paclitaxel *p<0.05.

As shown in Figure 3A, primary tumor growth was not affected by salinomycin treatment, but was significantly delayed by paclitaxel (p<0.05, at day 10 post-drug administration) compared with vehicle-treated animals. The combination of both therapies did not improve the effect of paclitaxel alone. Mice treated with salinomycin displayed a significant reduction in the number of lung macrometastatic foci compared with vehicle-treated mice (p<0.05; Figure 3B and C). Strikingly, mice treated with paclitaxel showed a higher number of macrometastasis compared with salinomycin-administered animals (p<0.05; Figure 3B and C). The number of metastatic foci in control mice was also lower than that found for paclitaxel-administrated mice, although no statistical differences were reached (Figure 3B). Values for the combination paclitaxel+salinomycin were in between single treatments alone, with no significant differences among them (Figure 3B). Further quantification of lung metastasis by image analysis in histological sections confirmed that administration of paclitaxel increased the ratio of tumor area/normal lung area compared to control animals (Figure 3C). These results show that the CSC-targeting drug salinomycin reduces metastasis but does not affect the primary tumor, whereas paclitaxel, although reducing primary tumor volume could foster metastatic spread. Results also show that, at least in this experimental model, the combination of salinomycin and paclitaxel is not an appropriate therapeutic option.

**Figure 4:**
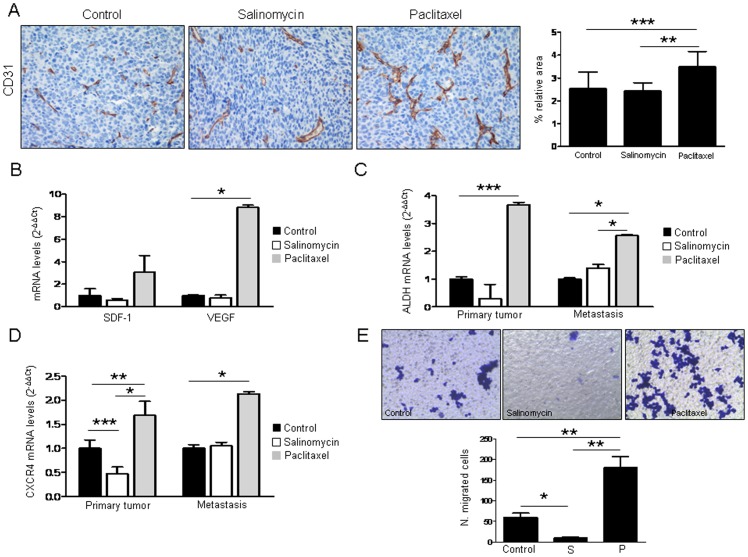
Expression analysis of CSC and angiogenesis markers. **A.** Quantification of CD31 in primary tumors. Measurements are given as relative area occupied by positive signals with respect to reference area. Significant increase in CD31 is found in paclitaxel treated mice. Representative IHC images of CD31 immunostaining (200x) in tumor samples are shown. **B.** Treatment with paclitaxel (40 ng/mL for 72 h) increases SDF-1 and VEGF levels in LLC cells. **C.** ALDH1A1 mRNA levels measured by qRT-PCR in primary tumors and metastatic nodules from mice treated with the drugs. Paclitaxel increases the expression of ALDH1A1 in both locations. **D.** CXCR4 mRNA levels measured by qRT-PCR in primary tumors and metastasis from control and treated mice. Paclitaxel increases the expression of CXCR4 in primary tumors and metastatic nodules. Salinomycin reduces the expression of CXCR4 in primary tumors but not in metastasis. **E.** Boyden chamber assays using LLC cells after treatment with either vehicle, salinomycin (1 µg/mL) or paclitaxel (40 ng/mL). A very significant increase in migration is observed in cells exposed to paclitaxel, whereas a significant reduction is found in salinomycin treated cells. *p<0.05. ***p<0.001. All the *in vitro* experiments were repeated at least 3 independent times (each of them in triplicates).

Analysis of primary tumor vascularization by means of CD31 quantification showed no difference between controls and salinomycin-administered mice. Surprisingly, tumors from animals that received paclitaxel were more vascularized (p<0.001) than controls (Figure 4A). In agreement with these results, we found that paclitaxel increased the expression of proangiogenic factors SDF-1/CXCL12 and VEGF (p<0.05) in LLC cells (Figure 4B). These data show, in agreement with the increased number of metastatic nodules, that paclitaxel modifies tumor stroma making it more favorable for angiogenesis and tumor spread.

We were intrigued by the fact that paclitaxel could enhance metastasis and angiogenesis. Our *in vitro* results demonstrated that paclitaxel increased the number of ALDH+ CSCs in several lung cancer cell lines. Therefore, we sought to investigate whether this drug would also increase ALDH levels *in vivo*. qRT-PCR analysis demonstrated that the expression of ALDH in both primary tumors (p<0.001) and metastatic lesions (p<0.05) was significantly higher in those mice that received paclitaxel, compared to controls, whereas animals administered with salinomycin had no significant changes (Figure 4C). Therefore, paclitaxel could be selecting the CSC population by allowing survival and expansion of these cells, which might be responsible for lung metastasis.

### Paclitaxel Induces CXCR4 and SDF-1 Expression in Primary Tumors and Metastasis

The CXCR4/CXCL12 chemotactic gradient has been proposed as a mechanism for tumor cell homing to distant metastatic sites [15]. Because lung cancer cells express CXCR4 (CD184), and stromal cells within the tumor microenvironment constitutively secrete SDF-1, which is the ligand for CXCR4 [26], we asked whether activation of the CXCR4/SDF-1 axis could be involved in migration to the metastatic site and drug resistance in our model. Levels of CXCR4 and SDF-1 were measured by qRT-PCR in primary tumors and metastases in both treated and control animals. Figure 4D and Figure S3A show that CXCR4 and SDF-1 were significantly elevated in both locations by paclitaxel treatment. On the contrary, such levels were reduced (or in some cases did not change) when mice administered with salinomycin. In order to confirm these *in vivo* results, CXCR4 expression was analyzed by flow cytometry after treatment of LLC cells with either vehicle, salinomycin (1 µg/mL) or paclitaxel (40 ng/mL). As shown in Figure S3B paclitaxel increased the expression of CXCR4 in LLC cell line (p<0.05) whereas salinomycin had an opposite effect (p<0.05). To investigate whether cells would have a different migratory ability upon drugs exposure, Boyden chamber assay was performed. As shown in Figure 4E salinomycin treatment inhibited cell migration (p<0.05) whereas an opposite result was found for paclitaxel (p<0.01). These results support our previous findings showing that paclitaxel may facilitate migration and homing of tumor cells to the metastatic site.

CSCs are thought to be responsible for metastasis, at least in part because they acquire a migratory phenotype [27], and secrete a large amount of cytokines that can alter the tumor microenvironment [28]. To analyze whether CSCs would be enriched in the CXCR4/SDF-1 migratory axis, levels of these markers were measured by qRT-PCR in LLC-derived spheres or adherent cells. As shown in Figure S3C we found that the expression of CXCR4 was significantly increased (p<0.01) in spheres compared to cells grown in adherent conditions, but no changes in SDF-1 expression were found. Therefore, CXCR4+ CSCs are more likely to be recruited to the lung by SDF-1 than the non-CSC population.

### Effect of Salinomycin and Paclitaxel on Primary Tumor and Metastatic Immune Cells of the Microenvironment

**Figure 5:**
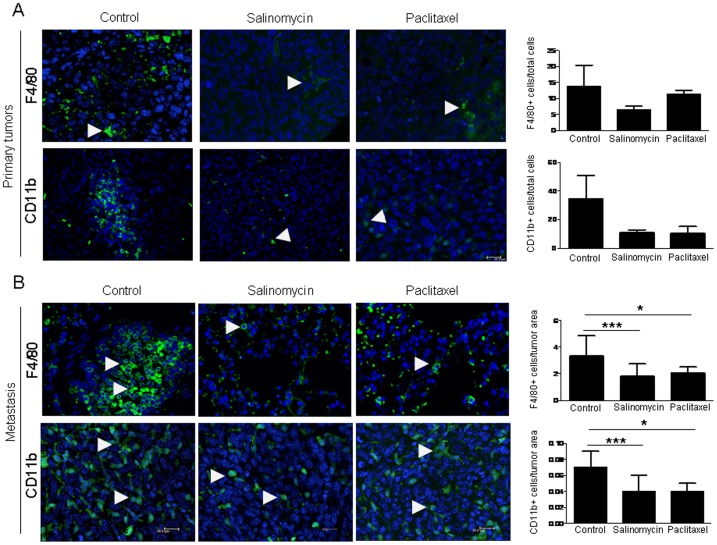
Staining and quantification of inflammatory cells in primary tumors and lung metastasis from salinomycin and paclitaxel treated mice. **A.** Immunofluorescence to detect F4/80+ or CD11b+ inflammatory cells. Arrowheads in the images indicate the positively stained cells. Both drugs reduce the number of F4/80+ and CD11b+ cells in primary tumors, but no statistical differences compared to controls are obtained. **B.** Staining and quantification in metastatic lesions. Significantly lower number of F4/80+ and CD11b+ cells are found in treated animals. Scale bar = 20 µm. Data are expressed as mean ± SEM. S: Salinomycin. P: Paclitaxel *p<0.05. ***p<0.001.

Increasing evidence shows that the behavior of tumorigenic cells is also highly influenced by their microenvironment. In addition, CSCs secrete numerous cytokines and growth factors that may alter the cell population within the tumor microenvironment. Because our previous results showed that salinomycin and paclitaxel had a different effect on CSCs and degree of lung colonization, which may involve changes in tumor microenvironment, we analyzed the effect of these drugs on inflammatory cells. The profuse number of F4/80 positive cells (macrophages) and CD11b positive cells (monocytes/macrophages) observed in both primary tumors and metastatic nodules of control mice were reduced by both drugs (Figure 5A and B), but differences reached statistical significance only in metastasis (Figure 5B). The effect of both drugs in reducing the number of F4/80+ and CD11b+ cells was similar. Neither paclitaxel nor salinomycin showed any influence on mast cell recruitment (Figure S3D).

### Drug Treatment Modifies the Different Expression Levels of CSC and Tumor Stroma-associated Markers in Primary Tumor and Metastatic Nodules

Results found throughout this study suggested different drug sensitivity between primary tumor and metastasis that could probably reflect a peculiar tumor cell behavior and interaction between tumor and stromal cells, depending on the tumor stage. To address this hypothesis, we analyzed the expression levels of CSCs markers ALDH and SOX2 and tumor stroma-associated markers CXCR4, SDF-1, VEGFR1, CD11b and α-sma in primary tumor and metastatic nodules. In control mice, we found that metastatic nodules expressed significantly higher levels of CSC (ALDH, SOX2) markers, CXCR4/SDF-1, and α-sma than primary tumors (Table 2). The expression of markers related to the establishment of the pre-metastatic niche (CD11b and VEGFR1) was also higher in metastatic lesions than in primary tumors. In mice administered with salinomycin or paclitaxel, a similar tendency was observed, with a higher expression of CSCs markers and stromal associated markers in the metastatic site than in primary tumors (Table 2). Of note, expression levels of some markers (such us ALDH, CXCR4 and VEGFR1) were much lower in salinomycin-treated animals than in control or paclitaxel-treated mice.

**Table 2:** Comparison of mRNA levels of CSCs related genes and tumor stroma associated markers in primary tumors and lung metastasis in control mice, and animals treated with salinomycin or paclitaxel.

A. Control mice.			
	PRIMARYTUMOR	METASTASIS	p value
**CONTROLS**			
**ALDH**	0.0012±0.09	4.59±0.53	0.007**
**SOX2**	0.000233±0.1	0.008±0.04	0.0350*
**CXCR4**	0.01±0.07	0.19±0.07	0.03*
**SDF1**	0.0007±0.006	0.06±0.01	0.021*
**VEGFR1**	3.61±0.76	10.54±0.95	0.008**
**CD11b**	0.063±0.011	0.426±0.075	0.0357*
**α-sma**	0.0056±0.34	3.67±0.47	0.0232*
**SALINOMICIN**			
**ALDH**	0.000078±0.0046	0.91±0.077	0.01**
**SOX2**	0.00053±0.41	0.009±0.02	0.025*
**CXCR4**	0.007±0.026	0.021±0.1	0.04*
**SDF1**	0.00023±0.06	0.021±0.2	0.054 ns
**VEGFR1**	0.00036±0.121	0.1±0.02	0.008**
**CD11b**	0.025±0.93	0.13±0.20	0.11 ns
**α-sma**	0.0034±0.01	0.96±0.16	0.005**
**PACLITAXEL**			
**ALDH**	0.05±0.03	5.79±0.49	0.0003***
**SOX2**	0.091±0.2	0.9±0.11	0.03*
**CXCR4**	0.011±0.02	0.59±0.036	0.025*
**SDF1**	0.0012±0.08	0.09±0.006	0.001***
**VEGFR1**	3.8±0.38	10.07±0.82	0.0002***
**CD11b**	0.088±0.61	0.26±0.032	0.0036**
**α-sma**	0.01±0.064	3.65±0.12	0.0012**

Five mice per group were used. Data are given as 2^−ΔCt^ for each gene with respect to GAPDH.

* p<0.05.

** p<0.01.

*** p<0.001.

ns: non significant.

## Discussion

In the present study we have investigated the different effects elicited by a drug that targets CSCs (salinomycin) and a conventional chemotherapeutic drug (paclitaxel) targeting non-CSCs, in both primary tumors and metastasis, using a syngeneic mouse model of metastatic lung cancer.

Our first task was to characterize the CSC population in the LLC cell line, since such information was lacking in the literature. We have demonstrated here that LLC cells contain an ALDH+ cell fraction (∼11%) that possesses typical traits of CSC, such as self-renewal capacity, increased tumorigenicity *in vivo*, resistance to chemotherapy, and the ability to give rise to tumorspheres when cultured in anchorage-independent conditions. These results are in agreement with previously published findings in human NSCLC cells where ALDH+ CSCs have been characterized [23]. The ALDH enzymatic activity has been described as a CSC marker in a variety of tumors (including lung), and expression of this protein in patients with lung and breast cancer is significantly associated with poor prognosis [5], [7].

Our experimental plan continued with the demonstration that salinomycin targets the ALDH+ population in lung cancer cells. Salinomycin was recently identified as a result of a screening process using a library of chemical compounds with a highly selective inhibitory effect on human breast CSCs *in vitro* [13]. Although the precise mechanism by which this compound exerts an antitumor activity is not yet clear, increasing numbers of studies are confirming its selectivity to target CSCs and are providing mechanistic data. Salinomycin is a potent inhibitor of the multidrug resistance P-glycoprotein (P-gp/MDR1/ABCB1) [14]. Fuchs *et al.* have shown that this compound causes programmed cell death in cancer cells by induction of apoptotic pathways [29]. In prostate CSCs, salinomycin reduces ALDH activity and the number of CD44+ cells [30]. Moreover, it has been shown to reduce Myc, AR and Erg levels, to inhibit nuclear factor-κB activity and to trigger oxidative stress in this type of cancer [30]. Salinomycin reduces the ALDH+ CSC population in gastric cancer cells [31] and inhibit tumorsphere formation and expression of CSC markers in the A549 lung cancer cell line as well [32].

Contrary to observations found with salinomycin in our study, paclitaxel enriched the CSC population, both *in vitro* and *in vivo*, particularly in metastatic lesions. This result was verified in our study in H460 and H1299 NSCLC cell lines. Our data validates the hypothesis that conventional chemotherapy selects for the CSC population in lung cancer. Previous studies have shown similar results in lung cancer cells [33] and other cancer types. In oral squamous cell carcinoma, both *in vitro* and *in vivo* treatment with paclitaxel resulted in a marked enrichment in CD133+ CSCs [34]. In paclitaxel-resistant ovary cancer cells, an increased side population (reflecting the CSC fraction) was observed compared to that found for the parental paclitaxel-sensitive cells [35]. In a series of 108 patients with breast cancer treated with neoadjuvant chemotherapy consisting of a sequential paclitaxel and epirubicin-based schedule, the proportion of ALDH+ CSCs increased significantly, as compared to untreated tumors [36] Lin *et al*. have recently demonstrated that cisplatin selects for CD133+ lung cancer stem cells by activating notch signaling. Moreover, these authors show that the number of CD133+ cells dramatically increase in NSCLC patients treated with cisplatin. All these data suggest that the CSC population may remain intact after chemotherapy, which would have biological and clinical implications in tumor resistance, recurrence and metastatic spread.

Although co-targeting both non-CSC and CSC populations with specific drugs did not improve the therapeutic efficacy compared to single drugs in our model, this hypothesis could still be valid for other drug combinations. In fact, salinomycin has been shown to synergize with gemcitabine to impair tumor growth in a model of pancreatic cancer [34]. A striking observation in our study was that treatment with paclitaxel, although reducing primary tumor volume, increased significantly the number of metastatic nodules, angiogenesis (and VEGF and SDF-1 levels in tumor cells) and expression of ALDH, SOX2, CXCR4 and SDF-1 *in vivo*. A similar unexpected prometastatic finding was recently described for VEGF-targeting antiangiogenic drugs (bevacizumab and sunitinib) in animal models [37], [38]. In this case, it was suggested that short-term administration of antiangiogenic therapy, though inhibiting primary tumor growth, could cause microenvironmental changes that are “conditioned” to be more permissive to tumor extravasation [37]. Such “conditioning” might imply overexpression of SDF-1, osteopontin and G-CSF, or the mobilization of bone marrow-derived cells [37].

Interestingly, a recent report by Gingis-Velitski *et al.* [39] revealed that plasma from animals treated with paclitaxel induced angiogenesis, migration and invasion of tumor cells *in vitro*, whereas gemcitabine caused much less of these effects. Moreover, authors demonstrated that paclitaxel strongly raised MMP-9 levels, induced EMT properties and accelerated metastasis and mice’s death in a metastatic model of intravenous injection of LLC cells [39]. Accelerated metastasis and death were not observed when animals received multiple cycles of paclitaxel, suggesting that long-term chemotherapy may overcome the protumorigenic response of the host [39]. The clinical benefit of paclitaxel in patients with lung cancer and other solid tumors has been clearly shown in many studies [40], but these results in preclinical studies shed light on the host acute response to chemotherapy and, in particular, to paclitaxel. These results may also explain in part the acquisition of drug resistance leading to tumor recurrence and progression in patients.

In our study, the fact that paclitaxel exerts a prometastatic effect would likely be associated to the relative increase in the proportion of the CSCs within the primary tumor upon treatment. It is reasonable to think that this enrichment would modify the CSC niche to facilitate the escape of these cells to distant organs. CSCs display EMT-like properties, such as high motility and invasiveness [27], [41], secrete high amount of proangiogenic cytokines [28] and are usually located nearby tumor blood vessels [42]. Therefore, perturbation of the CSC niche by an abnormal high CSC density may result in a promotion of their evasion from the primary tumor. We have shown here that CSCs express high levels of CXCR4 and that paclitaxel -unlike salinomycin- increases CXCR4 and SDF-1 levels in both primary tumors and metastasis. The CXCR4/CXCL12 (SDF-1) chemokine axis plays a key role in normal and malignant cell migration and homing, as well as in CSC biology [16]. Both CXCR4 and SDF-1 are significantly increased in NSCLC and expression of CXCR4 is associated with poor prognosis [43]. Moreover, blockade of CXCR4 with monoclonal antibodies or targeted drugs reduces cancer burden and metastasis [26]. The antimetastatic role of salinomycin observed in the present work can also be related, on the contrary to the fact that this compound reduces expression of CXCR4, SDF-1 and ALDH, thus impairing the CSC migratory potential. We can speculate that the one important effect elicited by salinomycin *in vivo* may be related to chemokine- or cytokine-mediated CSC migration to distant sites.

In summary, we have demonstrated that salinomycin targets the CSC population in NSCLC and plays a role in reducing the metastatic burden and decreasing expression of ALDH, SOX2, CXCR4, and SDF-1, as well as the number of CD11b+ and F4/80+ cells. This drug warrants further studies to address its mechanism of activity and its efficacy in clinical settings. Paclitaxel on the contrary, raises ALDH, CXCR4, and SDF-1 levels, and promotes metastatic spread (while reducing primary tumor volume) in the *in vivo* model we have used. Future work should investigate whether targeting CSC with specific drugs could be of clinical benefit in patients.

## Supporting Information

Figure S1 **A.** Representative FACS analysis for 7AAD exclusion in LLC cells. **B.** Purity of ALDH+ and ALDH− cells after sorting. **C.** ALDH+ and ALDH− LLC cells present similar growth rate. **D.** Percentage of ALDH+ cells in LLC-derived spheres. **E.** ALDH+ cells population in LLC-derived spheres after incubation with vehicle (20% DMSO as control) or paclitaxel (40 ng/ml). All the experiments were repeated at least three times (in triplicates).
(TIF)

Figure S2 **A.** ALDH mRNA levels measured by qRT-PCR in LLC cells treated with either vehicle salinomycin (1 µg/ml) or paclitaxel (40 ng/ml). ALDH expression is elevated upon administration of paclitaxel; salinomycin reduces ALDH mRNA levels. **B.** Representative FACS analyses for the ALDH+ population in the treated or untreated H460 cell line. **C.** ALDH mRNA levels measured by qRT-PCR in the human lung cancer cell lines H460 and H1299 treated either with either vehicle, salinomycin (1 µg/ml) or paclitaxel (40 ng/ml) for 72h. Expression of ALDH is highly increased by paclitaxel, whereas salinomycin reduces levels of this CSC marker. **D.** SOX2 mRNA levels in LLC cells (qRT-PCR). Paclitaxel increases the expression of this CSC marker. **E.** SOX2 expression in the human lung cancer cell lines H460 and H1299 treated either with either vehicle, salinomycin (1 µg/ml) or paclitaxel (40 ng/ml) for 72 h. Salinomycin reduces levels of SOX2. **F.** Sphere formation assay, with or without drugs (1 µg/ml salinomycin or 40 ng/ml paclitaxel). Salinomycin dramatically reduces the sphere formation ability of H460 and H1299 cells, whereas paclitaxel does not. Data and error bars are presented as mean ± SD. *p<0.05. **p<0.01. ***p<0.001. All the experiments were repeated at least three times (in triplicates).
(TIF)

Figure S3 **A.** SDF-1 mRNA levels measured by qRT-PCR in primary tumors and metastasis from control and treated mice. Paclitaxel increases the expression of SDF-1 in primary tumors and metastatic nodules. Salinomycin reduces the expression of SDF-1 in primary tumors but not in metastasis. **B.** FACS analysis for CXCR4 expression in LLC treated cells. Paclitaxel treatment increases CXCR4 expression whereas salinomycin has a opposite effect. **C.** Expression of CXCR4 and SDF-1 in LLC-derived spheres. CXCR4 levels are significantly increased in spheres compared to cells grown in adherent conditions. **D.** Toluidine blue staining to detect and quantify mast cells in tissue sections obtained from both primary tumors and metastatic nodules in mice treated with vehicle (controls), salinomycin or paclitaxel. Quantifications reveal no changes in the mast cell populations upon treatment with the drugs, as compared to controls. Data are expressed as mean ± SD or mean ± SEM for Figure D. *p<0.05. **p<0.01. ***p<0.001. *In vitro* experiments were repeated at least three times (in triplicates).
(TIF)

Table S1 List of Primers.
(DOC)
