# Supplementary figures and images for: Differential Effects of Drugs Targeting Cancer Stem Cell (CSC) and Non-CSC Populations on Lung Primary Tumors and Metastasis
Source: PLoS One. 2013 Nov 20;8(11):e79798. doi: 10.1371/journal.pone.0079798 (PMC3835894; doi:10.1371/journal.pone.0079798)

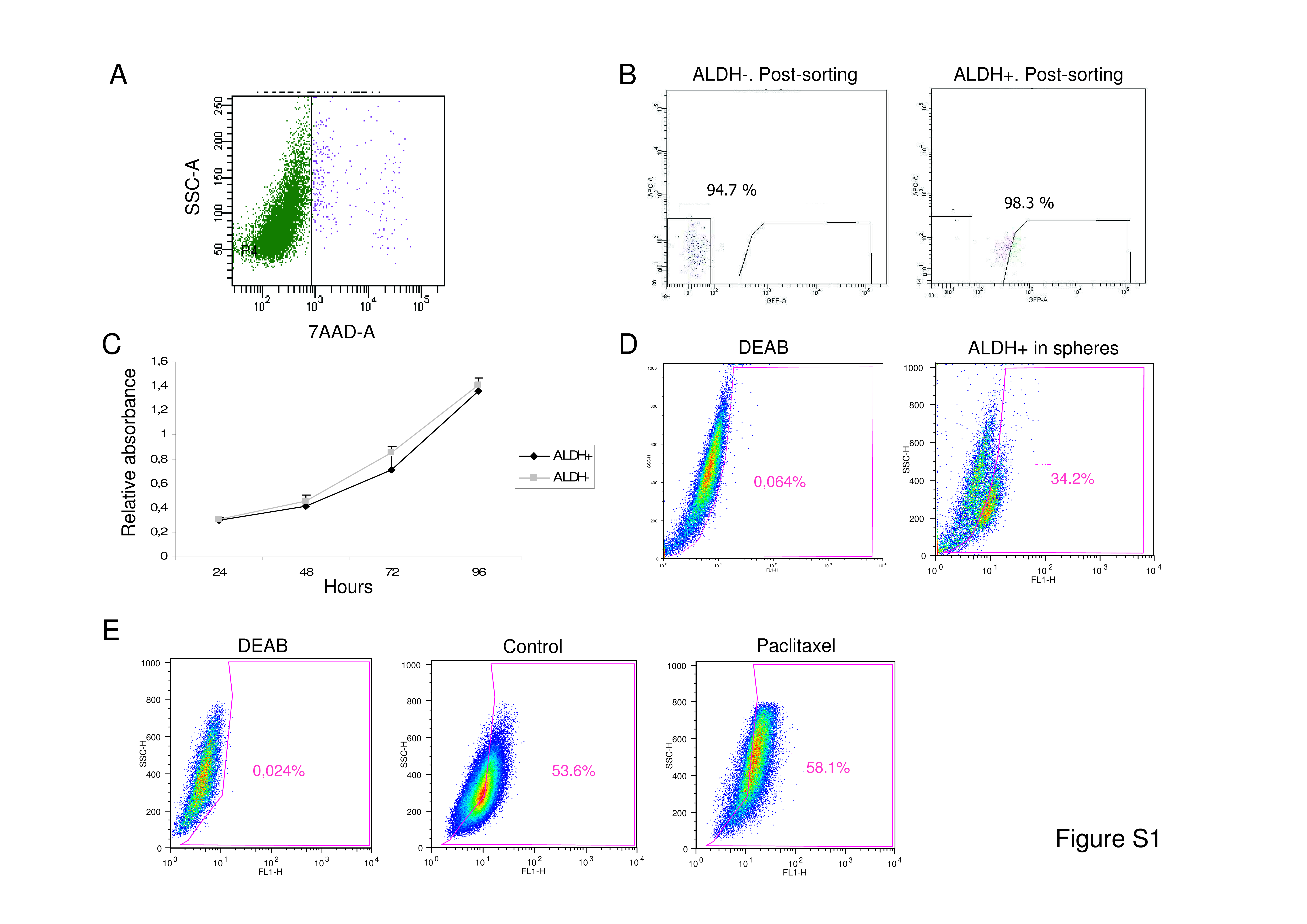

Supplement: Figure S1 — A. Representative FACS analysis for 7AAD exclusion in LLC cells. B. Purity of ALDH+ and ALDH− cells after sorting. C. ALDH+ and ALDH− LLC cells present similar growth rate. D. Percentage of ALDH+ cells in LLC-derived spheres. E. ALDH+ cells population in LLC-derived spheres after incubation with vehicle (20% DMSO as control) or paclitaxel (40 ng/ml). All the experiments were repeated at least three times (in triplicates). (TIF) [file pone.0079798.s001.tif]

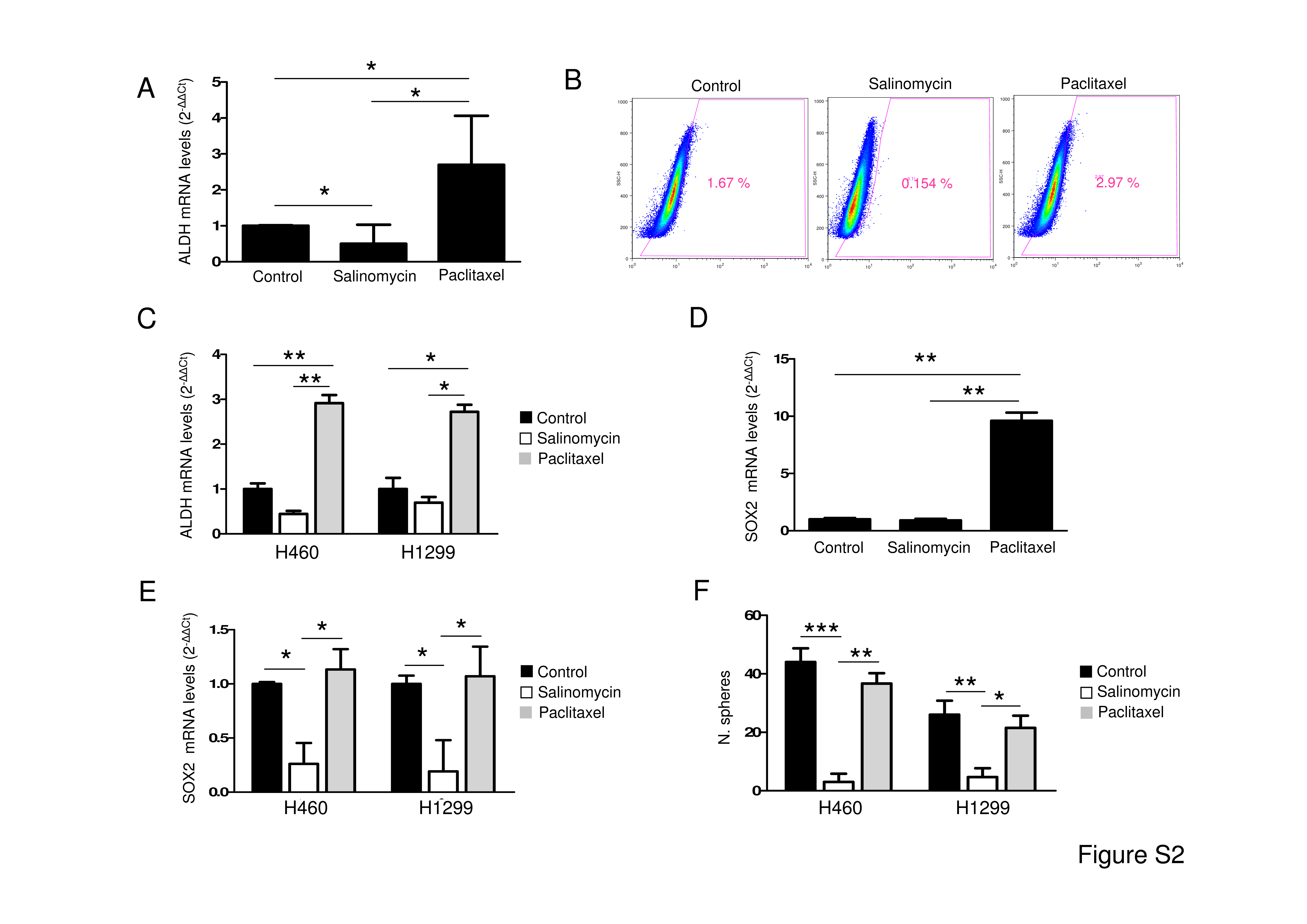

Supplement: Figure S2 — A. ALDH mRNA levels measured by qRT-PCR in LLC cells treated with either vehicle salinomycin (1 µg/ml) or paclitaxel (40 ng/ml). ALDH expression is elevated upon administration of paclitaxel; salinomycin reduces ALDH mRNA levels. B. Representative FACS analyses for the ALDH+ population in the treated or untreated H460 cell line. C. ALDH mRNA levels measured by qRT-PCR in the human lung cancer cell lines H460 and H1299 treated either with either vehicle, salinomycin (1 µg/ml) or paclitaxel (40 ng/ml) for 72h. Expression of ALDH is highly increased by paclitaxel, whereas salinomycin reduces levels of this CSC marker. D. SOX2 mRNA levels in LLC cells (qRT-PCR). Paclitaxel increases the expression of this CSC marker. E. SOX2 expression in the human lung cancer cell lines H460 and H1299 treated either with either vehicle, salinomycin (1 µg/ml) or paclitaxel (40 ng/ml) for 72 h. Salinomycin reduces levels of SOX2. F. Sphere formation assay, with or without drugs (1 µg/ml salinomycin or 40 ng/ml paclitaxel). Salinomycin dramatically reduces the sphere formation ability of H460 and H1299 cells, whereas paclitaxel does not. Data and error bars are presented as mean ± SD. *p<0.05. **p<0.01. ***p<0.001. All the experiments were repeated at least three times (in triplicates). (TIF) [file pone.0079798.s002.tif]

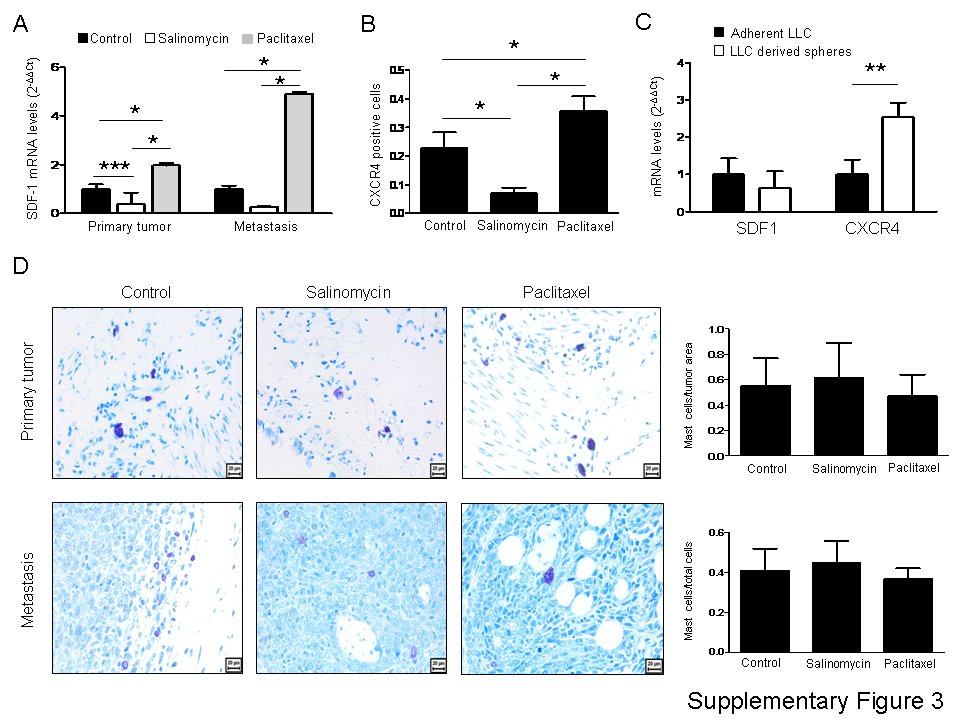

Supplement: Figure S3 — A. SDF-1 mRNA levels measured by qRT-PCR in primary tumors and metastasis from control and treated mice. Paclitaxel increases the expression of SDF-1 in primary tumors and metastatic nodules. Salinomycin reduces the expression of SDF-1 in primary tumors but not in metastasis. B. FACS analysis for CXCR4 expression in LLC treated cells. Paclitaxel treatment increases CXCR4 expression whereas salinomycin has a opposite effect. C. Expression of CXCR4 and SDF-1 in LLC-derived spheres. CXCR4 levels are significantly increased in spheres compared to cells grown in adherent conditions. D. Toluidine blue staining to detect and quantify mast cells in tissue sections obtained from both primary tumors and metastatic nodules in mice treated with vehicle (controls), salinomycin or paclitaxel. Quantifications reveal no changes in the mast cell populations upon treatment with the drugs, as compared to controls. Data are expressed as mean ± SD or mean ± SEM for Figure D. *p<0.05. **p<0.01. ***p<0.001. In vitro experiments were repeated at least three times (in triplicates). (TIF) [file pone.0079798.s003.tif]
