# Supplementary material for: Differential Effects of Drugs Targeting Cancer Stem Cell (CSC) and Non-CSC Populations on Lung Primary Tumors and Metastasis
Source: PLoS One. 2013 Nov 20;8(11):e79798. doi: 10.1371/journal.pone.0079798 (PMC3835894; doi:10.1371/journal.pone.0079798)
Supplement: Table S1 — List of Primers. (DOC) [file pone.0079798.s004.doc]

# ****Table S1: List and sequence of primers used in this work for real time PCR amplification.****

| GENE | SEQUENCE (5´->3´) |
| --- | --- |
| mouse ALDH1A1 | S GCACTCAATGGTGGGAAAGTAS GGCCACACACTCCAATAGGT |
| human ALDH1A1 | **S** TCCGGTTATGGGCCTACAG AS CTGGCCCTGGTGGTAGAATA |
| mouse SOX2 | S AACCCCAAGATGCACAACTCAS GAGCGTCTTGGTTTTCCGCC |
| human SOX2 | S AACCCCAAGATGCACAACTCAS CGGGGCCGGTATTTATAATC |
| mouse CXCR4 | S ACGGCTGTAGAGCGAGTGTTAS CCGTCATGCTCCTTAGCTTC |
| mouse SDF-1 | S GCCCTTCAGATTGTTGCACGGCAS GCCAGGGTTCAGGCCCTCCT |
| mouse VEGF | S GCGGATCAAACCTCACCAAAAS TTCACATCGGCTGTGCTGTAG |
| mouse VEGFR1 | S GAGGAGGATGAGGGTGTCTATAS GTGATCAGCTCCAGGTTTGAC |
| mouse CD11b | S TACGTAATTGGGGTGGGAAAS GTGCCCTCAATTGCAAAGAT |
| mouse α-sma | S CTGACAGAGGCACCACTGAAAS CATCTCCAGAGTCCAGCACA |
| mouse GAPDH | S ACTTTGTCAAGCTCATTCCAS TGCAGCGAACTTTATTGATG |
| human GAPDH | S ACTTTGTCAAGCTCATTCCAS CACAGGGTACTTTATTGATG |
